# Effect of Chin Tuck against Resistance Exercise in Citizens with Oropharyngeal Dysphagia—A Randomised Controlled Study

**DOI:** 10.3390/geriatrics7060129

**Published:** 2022-11-18

**Authors:** Diana Jensen, Bettina Burgdorff Bendsen, Signe Westmark, Johannes Riis, Anne Lund Krarup, Albert Westergren, Dorte Melgaard

**Affiliations:** 1 Centre of Rehabilitation, Municipality of Toender, 6270 Toender, Denmark; 2 Department of Physiotherapy and Occupational Therapy, Municipality of Hjoerring, 9800 Hjoerring, Denmark; 3 Centre for Clinical Research, North Denmark Regional Hospital, 9800 Hjoerring, Denmark; 4 Department of Geriatric Medicine, Aalborg University Hospital, 9000 Aalborg, Denmark; 5 North Denmark Regional Hospital, Bispensgade 37, 9800 Hjoerring, Denmark; 6 Department of Acute Medicine and Trauma Care, Aalborg University Hospital, 9000 Aalborg, Denmark; 7 Faculty of Clinical Medicine, Aalborg University, 9000 Aalborg, Denmark; 8 The PRO-CARE Group, The Research Platform for Collaboration for Health, Faculty of Health Sciences, Kristianstad University, 29128 Kristianstad, Sweden

**Keywords:** swallowing difficulties, eating difficulties, dysphagia, oropharyngeal, swallowing disorder, training

## Abstract

Oropharyngeal dysphagia (OD) impacts the safety and efficacy of the swallowing function. The aim was to uncover the effect of chin tuck against resistance (CTAR) exercises compared to standard care in relation to the swallowing function in citizens with OD. Ninety-two citizens (46% male, median age 78 years (IQR 71, 84)) with OD confirmed by the Volume-Viscosity Swallow Test and/or Minimal Eating Observation Form version II were randomised to standard care with the addition of CTAR daily for six weeks or standard care only. The participants were included from seven Danish municipalities from March 2019 to October 2020. A nonsignificant effect on dysphagia of CTAR training combined with standard care versus standard care alone was documented. Both CTAR training combined with standard care and standard care alone had a significant effect on the swallowing function in citizens with OD, with the best effect in the group receiving CTAR training combined with standard care. A significant effect compared to baseline was observed in all participants (*p* = 0.03) after 12 weeks. Participants in both groups had a significant reduction in problems with manipulating food in the mouth (*p* = 0.005), swallowing (*p* = 0.005), and chewing (*p* = 0.03) but an increased appetite (*p* = 0.01). The reported quality of life scored with DHI-DK was significantly improved in both groups.

## 1. Introduction

Oropharyngeal dysphagia (OD) is swallowing difficulties arising in the oral cavity or pharynx. OD may be caused by structural alterations affecting food bolus progression, which impacts the safety and efficacy of the swallowing function [1,2,3]. The prevalence of OD is >50% in patients with, for example, Parkinson’s disease, stroke, cancer, and dementia, as well as among institutionalised older people [4]. OD results in multiple episodes of pneumonia, malnutrition, dehydration, and depression, as well as reduced quality of life (QoL), rehospitalisation, and death [3,5,6].

In Denmark, the rehabilitation of dysphagia primarily takes place in the citizen’s homes with the assistance of municipal health professionals. Citizens referred for municipal dysphagia rehabilitation often also have a high rate of comorbidity.

Tongue pressure and suprahyoid muscle function play an essential role in the swallowing process and the protection of the airway [7]. The suprahyoid muscles are the primary muscles involved in the pharyngeal phase with an elevation of the larynx [8]. Reduced elevation of the larynx has been associated with aspiration [9]. Strengthening of the suprahyoid muscle function is, therefore, important in dysphagia rehabilitation. Exercises strengthening the tongue and suprahyoid muscles can be used to treat OD; the two most common are the Shaker exercise and the Chin Tuck Against Resistance (CTAR) exercise. A study showed that the CTAR exercise was more effective compared to the Shaker exercise [7].

Several studies including healthy participants have documented that the CTAR exercise could increase the power of the suprahyoid and sternocleidomastoid muscles [10,11,12,13]. A systematic review from 2021 showed that the CTAR exercises could improve swallowing function in patients with stroke-induced OD [8].

The present study aimed to evaluate the effect on the swallowing function in citizens with OD by adding the CTAR exercise to standard care and comparing the results to citizens receiving standard care only.

## 2. Materials and Methods

**Figure 1:**
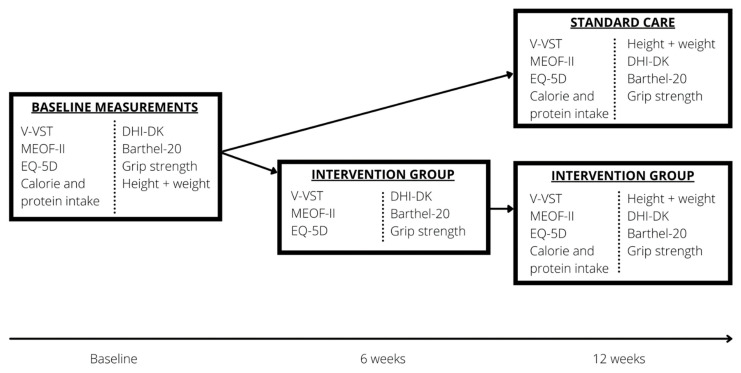
The study protocol. VVST: Volume Viscosity Swallow Test, MEOF-II: Minimal Eating Observation Form-Version II, EQ-5D: EuroQol-5 Domain, DHI-DK: Dysphagia Handicap Index—DK.

This randomised controlled study was conducted from March 2019 to October 2020. It was approved by the North Denmark Committee on Health Research Ethics (N-20180061), registered at the Danish Data Protection Agency (2008-58-0028) and ClinicalTrials.gov (NCT04402307). Participants from seven geographically spread Danish municipalities (Hjørring, Frederikshavn, Jammerbugt, Kolding, Odense, Tønder, and Brøndby) were included. Informed consent was obtained from all participants. Participants were randomised 1:1 to receive either (1) standard care or (2) standard care with the addition of CTAR training. All participants filled out questionnaires at baseline and 12 weeks after inclusion. Furthermore, the intervention group filled out the questionnaires after six weeks of CTAR training (Figure 1).

### 2.1. Participants

Inclusion criteria were: +18 years, OD confirmed by Volume-Viscosity Swallow Test (V-VST) and/or Minimal Eating Observation Form version II (MEOF-II), ability to give and understand informed consent. V-VST and MEOF-II were chosen to uncover the complexity of eating and drinking, and trained and experienced occupational therapists (OT) administered tests. Exclusion criteria were citizens with severe dementia or other cognitive impairments, as well as individuals receiving palliative care or using feeding tubes and citizens already referred for dysphagia training. Furthermore, participants hospitalised for more than seven days during the study period were excluded.

### 2.2. Tests

The V-VST assesses different types of viscosity (mineral water, nectar, and pudding) and volumes (5, 10, and 20 mL). Water was modified by adding the thickener Resource Thicken Up (Nestlé HealthCare Nutrition). Boluses of each volume and viscosity were administered to the citizens with a syringe. The following clinical signs of swallowing were also observed: changes in voice quality, cough, or decrease in oxygen saturation ≥3% to detect silent aspiration. One or more signs of impaired safety or efficacy indicated OD [9].

The MEOF-II measures eating performance. It consists of nine items in the following three categories: (1) Ingestion, (2) deglutition, and (3) energy. The eating difficulties can be summed, and the total score ranged from 0 to 9, with higher scores representing a higher level of dysfunction [10,11]. The citizens were observed eating a meal consisting of a range of different textures and viscosities, for example, breakfast with yoghurt, bread, apples, coffee, and juice.

Both tests were performed at baseline, at a six-week follow-up for the intervention group and at a 12-week follow-up for both groups, to measure the progression of OD.

### 2.3. Treatment Protocol

In Denmark, Standard care in municipal dysphagia rehabilitation consists of one to two contacts with an OT and a dietitian. The aim is to inform the citizen about dysphagia and risk factors, provide adjustments in sitting position, customise the texture of food and viscosity of beverages, and offer information about the importance of oral healthcare.

In the Intervention group, the OT explained and demonstrated the CTAR exercise program before the intervention. The participants were instructed to be seated in a chair with good support and feet placed on the floor and to place an inflatable ball (dia. 12 cm) between the chin and the sternum. The exercise consists of two parts: (1) a static part where the ball is compressed between the chin and the sternum, and pressure is held for 30 s while swallowing hard; (2) a dynamic part where the ball is compressed between the chin and the sternum 10–30 times until the neck muscles are tired. Both parts of the exercise were repeated three times a day for six weeks.

During the period of six weeks, the OT visited the participants twice a week to make sure the CTAR exercise was performed correctly.

### 2.4. Questionnaires

The standardised questionnaires and tests mentioned below measure health-related quality of life (HRQoL) in relation to OD, the ability to perform activities of daily living (ADL), and provide information on energy intake, hand grip strength and severity of OD.

The Dysphagia Handicap Index—Danish version (DHI-DK) is a diagnosis-specific questionnaire examining how citizens experience problems with swallowing. The questionnaire covers 25 areas with three possible answer categories: never, sometimes, or always. The severity of experienced OD was registered at an equal interval scale from 1–7, where 1 indicated ‘no difficulty at all’; 4 was ‘somewhat of a problem’, and 7 was ‘the worst problem you could have’ [12].

The Barthel 20 index is a generic tool assessing levels of functional disability and ADL. Citizens were assessed according to 10 basic activities and were scored on their dependence and need for assistance to perform the activity. This score is 0–3 points, and the maximum total score is 20; the higher the score, the higher the dependency [13,14].

The citizens scored their overall self-reported health condition from the ‘worst thinkable health condition’ to ‘best possible health condition’ on a visual analogue scale used in EQ-5D and rating from 0 to 100 J [15].

Nutritional screening was carried out by a dietitian. The dietitian registered the actual daily calorie and protein intake during an interview. Furthermore, the need for the right daily calorie and protein intake was calculated to guide the citizens concerning their dietary composition. Height and weight measurements were collected, and the Body Mass Index (BMI) was calculated.

The hand grip strength of the dominant hand was measured three times, and the average was calculated. Hand grip strength was measured with a Jamar dynamometer (G.E. Miller, Inc., 484 Broadway, Yonkers, New York, NY, USA, 10705) [16,17].

The Functional Oral Intake Scale (FOIS) reflects the functional oral intake of citizens with OD on a 7-point Likert scale; one indicates ‘no difficulty at all’, and 7 indicates ‘the worst problem you could have’ [18].

Prior to the study, all OTs and dietitians involved in the study participated in a two-day course on testing for OD and the collection of data.

### 2.5. Data

Study data were collected and managed using the Research Electronic Data Capture tool (REDCap) hosted at North Denmark Region [19,20]. REDCap is a secure, web-based software platform designed to support data capture for research studies. Participants were randomised 1:1 using REDCap either to the standard care group or intervention group.

### 2.6. Sample Size Estimation

Given that 95% of participants with a V-VST indicated OD at follow-up in the control group and 70% in the intervention group, we calculated a sample size of 35 in each group at 80% power and a significance level of 5%. Thus, we included 45 patients in each group due to an expected dropout during the 12-week study period.

### 2.7. Statistics

When reporting results, categorical data were presented using numbers and percentages and compared by Chi2 tests. Continuous data were presented using means and standard deviations (SD) or medians and interquartile ranges (IQR) if non-normally distributed. Continuous variables were compared by *t*-tests if normally distributed and by the Mann–Whitney Test if not.

For the intention to treat analysis, multiple imputation was performed of missing outcome data to reduce bias. We assumed that data were missing at random and used 20 imputations. All outcome variables (including baseline values) were used for the imputation. Analysis of primary outcomes (dysphagia tests) was performed using logistic regression adjusted for baseline values (except for the overall outcome of any test showing dysphagia, as 100% of the participants had this at baseline). Finally, we also presented absolute and relative values of outcome data among complete cases and compared them with baseline values in both groups combined using McNemar’s test. All calculations were performed in R version 3.5.3.

## 3. Results

**Figure 2:**
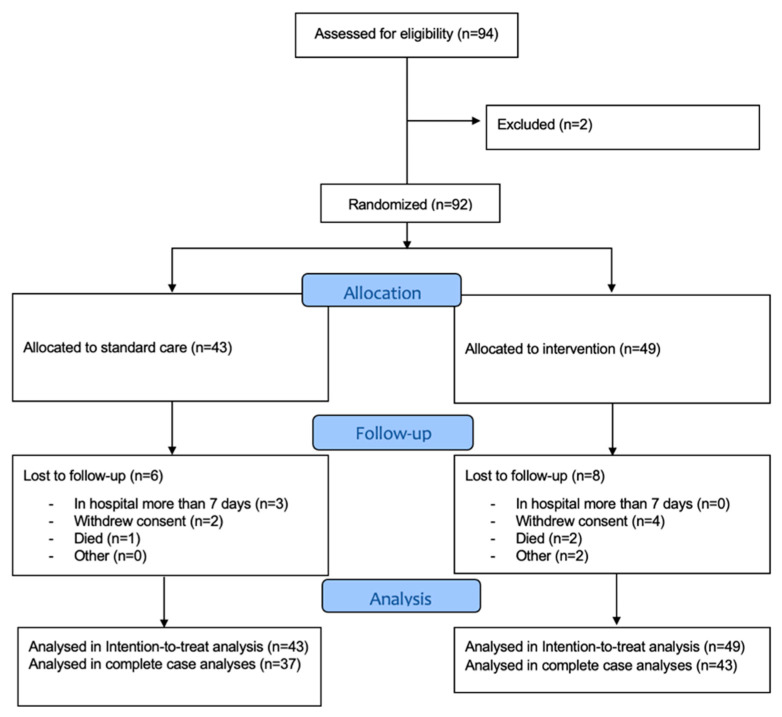
Flowchart for enrolment and inclusion of participants. Baseline characteristics of participants can be seen in Table 1. No systematic differences between participants were found.

Ninety participants were included between March 2019 and October 2020. The enrolment and inclusion of participants is shown in Figure 2.

There was a trend towards an effect of the CTAR training in the intervention group compared with the standard care group, although this was not statistically significant in the intention-to-treat analysis (OR 0.48, 95% CI 0.17 to 1.35, *p* = 0.16). The trend was also seen across different assessment methods for dysphagia (Table 2), and similar results were seen for secondary outcomes.

**Figure 3:**
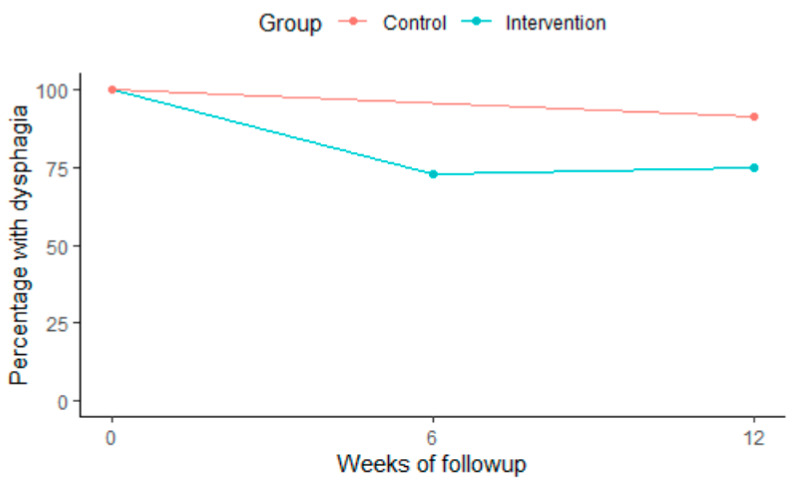
Percentage of participants with dysphagia over the study period among complete cases.

This positive trend in the intervention group was also seen when presenting the proportion of participants with OD in both groups among complete cases (Figure 3). All had OD at baseline, while 62.3% (*n* = 31) in the intervention group and 76.7% (*n* = 33) in the control group had dysphagia at follow-up after 12 weeks (*p* = 0.24 among complete cases). The absolute values of outcomes can be found in Table 2.

### Follow-Up

As shown in Table 3, about one-third of the participants showed no signs of OD as measured by the V-VST at 12 weeks following the six-week intervention with CTAR training and standard care compared with a reduction of one-fifth in the group receiving standard care only, also measured by the V-VST. The numbers were even higher when OD was measured with the MEOF-II. When comparing changes in all participants compared to baseline, there was a significant reduction in participants with problems manipulating food in the mouth (*p* = 0.03), swallowing (*p* = 0.005), and chewing (*p* = 0.03). Moreover, participants reported an increasing appetite (*p* = 0.01), and the energy to eat increased (*p* = 0.08). Protein intake did not significantly increase from baseline (*p* = 0.25); the intervention group had an increased protein intake of 3.1% versus 4.7% in the standard care group. The effect on BMI was limited. The reported QoL score with DHI-DK was significantly improved in both groups (mean increase 4.6, 95% CI: 1.4–7.8, *p* = 0.005).

All continuous data are reported as medians with interquartile range. BMI, body mass index; DHI, Dysphagia Handicap Index.

## 4. Discussion

The present study compared the effect on swallowing function after 12 weeks in participants with OD receiving standard care combined with CTAR training for six weeks and participants receiving standard care only. Standard care included supervision by an OT and a dietitian concerning the texture and viscosity of food and beverages, energy intake, adjustments in sitting position, information about OD, and risk factors. Swallowing function and HRQoL were considerably improved in both groups, and there was a trend, though not statistically significant, towards a better effect in the group receiving CTAR training combined with standard care.

Few studies on OD have documented general improvement in swallowing among participants receiving standard care. A hospital-based study documented a significant, positive effect on nutritional status, functionality, and reduced hospital readmissions, respiratory infections, and mortality. The intervention in this study included fluid thickening and texture-modified foods, calorie and protein supplementation, and verbal health advice during hospitalisation and after discharge [21].

The present study corroborates findings from a systematic review documenting an effect on swallowing function in patients with neurological diseases undergoing CTAR training [8]. None of the studies in the review compared the effect of CTAR training versus standard care, and no studies tested the effect in a group of patients with multimorbidity as in the present study.

In the present study, the ability to swallow decreased when the systematic training stopped after six weeks. It is well known that muscle strength and effect decrease when training stops [22]. After 12 weeks, there was still a positive effect of CTAR training combined with standard care versus standard care alone, but the effect is expected to decrease further over time. Thus, the best effect will probably be seen in those who can continue CTAR training after the 12-week intervention period.

The intervention in our study affected not only the swallowing function, but also other aspects of the eating and drinking activities. The complexity of the study sample in general and eating difficulties need to be considered when interpreting the findings. Conducting a municipality-based intervention study among persons experiencing OD related to a range of different diagnoses and comorbidities is demanding. Different factors may have interfered negatively with compliance in relation to the intervention in both the intervention group and the standard care group. The composition of the study sample, including participants with different diagnoses, means that participants had different disease courses. Persons with, for example, stroke, tend to improve their eating ability after the event [23], while persons with Parkinson’s disease tend to experience an increasing degree of eating problems as the disease progresses [24]. It is important for clinicians to be aware of OD among citizens with different diagnoses and comorbidities as they reflect the population mix in municipal rehabilitation settings. It is encouraging, however, to see the trend towards a positive effect on OD following CTAR training combined with standard care versus standard care only.

The study sample’s complexity relates not only to diagnosis and comorbidity but also to eating difficulties per se. Both groups in our study considerably improved their eating ability, including manipulating food in the mouth, swallowing, and chewing; moreover, both groups reported having more energy to eat and increased appetite. Thus, the intervention focused on the ability to swallow but also impacted other aspects of eating, highlighting that different parts of the eating activity are interconnected. This interconnectedness was illustrated in a study [25] showing how nine eating problems were hierarchically ordered. Our findings showed that improvements were mainly found in relation to energy to eat, appetite and manipulating food in the mouth, swallowing, and chewing. Improvements were not seen concerning ingestion (sitting position, manipulating food on the plate, and transporting food to the mouth). It is especially important that the energy to eat was improved, as this may have a major impact on the ability to eat, especially swallowing [26], and on the risk of developing undernutrition in some populations [10,25,26]. It is likely that the increased focus on the swallowing ability in both groups also increased the participants’ awareness of the overall eating situation. This may explain the improvements seen in other aspects of eating. Furthermore, since there were general improvements in the ability to eat in both groups, the possibility of detecting a difference specifically related to dysphagia was challenging. In clinical practice, it seems important not to only focus on one part of the eating activity, since these activities are considered interrelated.

It is well known that OD often leads to unplanned weight loss [3]. In our study, there was no significant change in BMI in either of the groups, and it is positive that the participants maintained their weight during the study period.

The randomised controlled design of this study is a strength, as well as the primary outcome being the participants’ ability to swallow. Unlike previous studies, participants with complex issues and multimorbidity have been included in the present study, and our results are directly transferable to clinical practice in municipal rehabilitation settings.

Recognised, standardised, and validated research methods exist in OD and nutritional assessment [27,28,29]. Instrumental measures are viewed as the gold standard in detecting dysphagia, but, in this study, it was not possible to perform instrumental examinations to uncover OD, as this is practically impossible in the citizen’s own home. Aspiration may be detected but not related to the efficacy of the eating situation; therefore, whether this is a limitation or a strength should be discussed.

It is a weakness that the participants’ cognitive and mental state is not uncovered, as it is known that there is a connection between cognition and dysphagia [30]. More than 30% of the participants had experienced a stroke, but information about stroke onset was unknown. The study period could have been prolonged to optimise the strength of the study.

## 5. Conclusions

This randomised controlled study demonstrates that standard care with a multi-professional approach, involving one or two visits by an OT and a dietitian combined with a six-week intervention with CTAR, improved the swallowing function and the HRQoL the participants maintained their weight compared with a group receiving standard care only. The multi-professional approach alone, which is standard care in most Danish municipalities, improved the swallowing function and the HRQoL as well: not as much as the intervention involving CTAR, but still significant compared with the baseline.

Based on this study, all citizens with OD should receive information about texture/viscosity, sitting position, and dietary composition. Citizens who can continue training after completing municipal rehabilitation will receive a good effect from instruction in CTAR.

## Figures and Tables

**Table 1:** Baseline characteristics of participants.

	Missing	Intervention Group *n* = 48	Standard Care Group *n* = 42	*p*-Value
Age (median, IQR)	0	79.0 (71.2, 84.4)	77.1 [71.0, 84.5]	0.77
Female sex	0	24 (51.1%)	24 (57.1%)	0.79
BMI	0			0.41
Underweight <18.5		8 (17.0%)	4 (9.5%)	
Normal weight 18.5–25		18 (38. 3%)	15 (35.7%)	
Overweight 25–30		10 (21.3%)	15 (35.7%)	
Obese >30		11 (23.4%)	8 (19.1%)	
EQ5D VAS 0–100	11	55 (40, 75)	50 (40 77)	0.56
Hand grip strength (kg)	3	15.1 (9.1, 26.2)	22.0 (12.9, 26.3)	0.14
History of stroke	0	17 (35.4%)	13 (33.3%)	>0.99
Other neurological comorbidity	0	7 (14.6%)	7 (16.7%)	>0.99
Respiratory comorbidity	0	18 (37.5%)	12 (28.6%)	0.50
Cardiac comorbidity	0	10 (20.8%)	17 (40.5%)	0.07
Ear, nose, and throat comorbidity	0	3 (6.3%)	1 (2.4%)	0.71
Rheumatological comorbidity	0	16 (33.3%)	12 (28.6%)	0.80
Other diseases	0	20 (41.7%)	18 (42.9%)	>0.99
FOIS score				0.25
Score 4	0	1 (2.08)	4 (9.52)	
Score 5	0	24 (50.00)	22 (52.38)	
Score 6	0	23 (47.92)	16(38.10)	
Living situation	47			0.45
Living independently		13 (56.5%)	11 (55.0%)	
Temporary in rehabilitation		5 (21.7%)	7 (35.0%)	
Nursing home resident		5 (21.7%)	2 (10.0%)	

Abbreviations: IQR, Interquartile range; BMI, body mass index; EQ5D, EuroQol-5 Dimension; VAS, Visual analogue scale; FOIS, The Functional Oral Intake Scale.

**Table 2:** Effect of intervention on overall frequency of dysphagia, V-VST and MEOF-II based on logistic regression with multiple imputation (*n* = 92).

	Intervention Effect Versus Standard Care? Odds Ratio for Test Indicating Problem at Follow-Up. Lower ORs Indicate a More Positive Result for the Intervention Group.	*p* Value
**Any Test Showing Dysphagia**	0.32 (95% CI: 0.07 to 1.17)	0.08
**Volume-Viscosity Swallow Test**	0.66 (95% CI: 0.24 to 1.84)	0.42
Impaired safety	0.67 (95% CI: 0.24 to 1.87)	0.44
Impaired efficacy	0.81 (95% CI: 0.31 to 2.15)	0.67
**Minimal Eating Observation Form-II**	0.80 (95% CI: 0.31 to 2.09)	0.65
*Ingestion*		
Sitting position	1.51 (95% CI: 0.33 to 6.80)	0.59
Manipulation of food on the plate	0.48 (95% CI: 0.14 to 1.65)	0.24
Transport of food to the mouth	1.53 (95% CI: 0.51 to 4.65)	0.44
*Deglutition*		
Manipulation of food in the mouth	0.74 (95% CI: 0.29 to 1.89)	0.53
Swallowing	0.76 (95% CI: 0.30 to 1.88)	0.55
Problems chewing (often/very often)	0.84 (95% CI: 0.26 to 2.73)	0.77
	0.95 (95% CI: 0.33 to 2.68)	0.91
*Energy*		
Energy to eat	1.00 (95% CI: 0.34 to 2.93)	0.99
Appetite (low/very low)	1.00 (95% CI: 0.34 to 2.93)	0.57
Amount of food (half a portion or less)	0.26 (95% CI: 0.06 to 1.09)	0.56

**Table 3:** Descriptive information on outcome data.

	Baseline		6 Weeks	12 Weeks	
	CTAR + Standard Care *n* = 48	Standard Care *n* = 42	CTAR + Standard Care *n* = 45	CTAR + Standard Care *n* = 40	Standard Care *n* = 35
**Volume-Viscosity Swallow Test**	45 (93.8%)	41 (97.6%)	24 (53.3%)	26 (65.0%)	28 (80.0%)
Impaired safety	40 (83.3%)	28 (66.6%)	18 (40.0%)	19 (47.5%)	22 (62.9%)
Impaired efficacy	29 (60.4%)	26 (61.9%)	17 (37.8%)	26 (65.0%)	21 (60.0%)
**Minimal Eating Observation Form-II**	42 (91.3%)	31 (77.5%)	25 (55.6%)	17 (42.5%)	14 (40.0%)
*Ingestion*					
Sitting position	8 (17.4%)	9 (22.0%)	8 (17.8%)	8 (20.0%)	6 (17.1%)
Manipulation of food on the plate	15 (32.6%)	13 (31.7%)	10 (22.2%)	9 (22.5%)	11 (31.4%)
Transport of food to the mouth	9 (19.6%)	13 (31.7%)	8 (17.8%)	7 (17.5%)	5 (14.3%)
*Deglutition*					
Processing? of food in the mouth	24 (52.2%)	16 (39.0%)	13 (28.9%)	10 (25.0%)	10 (28.6%)
Swallowing	32 (78.3%)	21 (51.2%)	14 (31.1%)	13 (32.5%)	14 (40.0%)
Problems chewing (often/very often)	10 (21.7%)	5 (12.2%)	3 (6.7%)	2 (5.0%)	1 (2.9%)
*Energy*					
Energy to eat	14 (30.4%)	7 (17.1%)	6 (13.3%)	6 (15.0%)	4 (11.4%)
Appetite (low/very low)	19 (41.3%)	17 (41.5%)	13 (28.9%)	10 (25.0%)	9 (26.5%)
	6 (13.0%)	4 (9.8%)	3 (6.7%)	3 (7.5%)	4 (11.4%)
Amount of food (half a portion or less)	*Missing = 2*	*Missing = 2*	*Missing = 0*	*Missing = 0*	*Missing = 0* *(appetite = 1)*
**Energy Intake** (kilojoule)	6635 (5425, 7532)	6663 (5222, 7705)	-	7216 (6057, 7947)	6700 (5992, 8250)
	*Missing = 10*	*Missing = 6*		*Missing = 18*	*Missing = 13*
**Protein Intake** (g/day)	54.4 (45.8, 70.2)	48.6 (41.5, 72.1)	-	63.0 (51.5, 75.0)	68.8 (47.5, 86.9)
	*Missing = 11*	*Missing = 6*		*Missing = 19*	*Missing = 13*
**BMI**	24.5 (20.5, 29.28)	25.6 (21.3, 29.2)	25.4 (20.0, 30.1)	25.2 (21.2, 29.3)	27.0 (22.5, 29.0)
	*Missing = 1*	*Missing = 2*	*Missing = 10*	*Missing = 1*	*Missing = 3*
**DHI**					
Total score	32 (22, 43)	24 (16, 32)	24 (12, 40)	19 (12, 37)	18 (8,34)
Physical score	14 (10, 18)	12 (8, 14)	10 (6, 16)	9 (8, 13)	8 (6, 16)
Emotional score	6 (2, 14)	4 (0, 8)	4 (0, 10)	4 (0, 10)	2 (0, 8)
Functional score	10 (6, 16)	8 (4, 10)	10 (2, 14)	6 (0, 13)	6 (2, 12)
	*Missing = 1*	*Missing = 1*	*Missing = 0*	*Missing = 0*	*Missing = 0*
**Barthel Index**	85 (50, 93)	80 (80, 95)	85 (55, 95)	90 (55, 95)	90 (75, 100)
	*Missing = 1*	*Missing = 1*	*Missing = 0*	*Missing = 1*	*Missing = 1*

## Data Availability

Data can be received by contacting corresponding author.
